# Alternative Pharmaceutical Innovation Models in Competitive Markets: A Collaborative Approach to Develop a Novel Drug for Hepatitis C

**DOI:** 10.3390/tropicalmed9100233

**Published:** 2024-10-08

**Authors:** Marcela Vieira, Iulia Slovenski, Kaitlin Large, Adrian Alonso Ruiz, Suerie Moon

**Affiliations:** Global Health Centre, Geneva Graduate Institute, 1205 Geneva, Switzerland; iulia.slovenski@graduateinstitute.ch (I.S.); kaitlin.large@graduateinstitute.ch (K.L.); adrian.ruiz@graduateinstitute.ch (A.A.R.); suerie.moon@graduateinstitute.ch (S.M.)

**Keywords:** pharmaceutical research and development, drug development, alternative innovation model, product development partnerships, access to medicines, hepatitis C, neglected diseases, ravidasvir

## Abstract

Alternative innovation models have emerged to address failures of the traditional pharmaceutical system, particularly for diseases where market incentives do not attract sufficient research and development efforts. However, the feasibility of such models for diseases with significant markets is not well-established. This article analyses the development of a novel drug (ravidasvir) for the treatment of hepatitis C, a highly profitable market. Data from qualitative research methods, including literature reviews and semi-structured interviews, was analyzed using a novel conceptual framework focusing on actors, resources, organizational practices, and outcomes. Dissimilar to other projects, ravidasvir did not involve any major pharmaceutical companies. Rather, it leveraged the capacities of actors less traditionally involved in the development of novel medicines by constructing a collaborative network of private and public partners from low- and middle-income countries with a shared goal. The collaboration was successful in developing a highly effective, easy-to-use, and affordable medicine and contributed significantly to capacity-strengthening. However, the case also highlighted that strategic behavior by competing for-profit firms could pose significant challenges and that changing external conditions reduced the potential public health impact of the drug. Lessons from ravidasvir can inform future efforts to develop alternative innovation models for therapeutic areas with significant commercial interest.

## 1. Introduction

The hepatitis C virus (HCV) poses a significant public health challenge globally. According to the World Health Organization (WHO), an estimated 50 million people are living with chronic hepatitis C infection worldwide, with about 1 million new infections occurring per year [1]. Despite advancements in prevention and treatment, hepatitis C remains a leading cause of liver-related morbidity and mortality, with an estimated 244,000 deaths in 2022 [1]. The WHO has outlined specific goals to eliminate viral hepatitis as a public health threat by 2030, including increasing the number of people diagnosed and treated [2]. Nevertheless, access to diagnosis and treatment is still low [3], and by the end of 2022, only 13.2 million people had been treated globally [4]. In this article, we analyze the innovation model that led to the development of a new treatment for hepatitis C aiming to address some of the access challenges, with a special focus on low and middle-income countries (LMICs).

The landscape of hepatitis C management has undergone a remarkable transformation in recent years. The development of several direct-acting antiviral (DAA) therapies has revolutionized treatment outcomes since the mid-2010s, with easy-to-use all-oral regimens, high cure rates (>95%), and relatively minimal side effects [5]. Previously, the standard of treatment was injected interferon-based regimens, with success rates between 40 and 70% and severe side effects [6]. However, when they were first launched, DAAs were very expensive, restricting access for patients.

HCV is classified into genotypes 1 through 6, and each may respond differently to specific antiviral medications. Globally, the two most prevalent genotypes of HCV are genotype 1, which is more prevalent in HICs, and genotype 3, which is more prevalent in LMICs [7,8]. Genotypes that are less prevalent in HICs have not been fully assessed in clinical trials [9]. Furthermore, they also presented the risk of drug–drug interactions, particularly with HIV drugs, leaving some populations without well-proven treatment options [7,10,11]. Finally, there is a risk of the virus becoming resistant to existing treatments [9] and, therefore, potentially a need for more treatment options.

In addition to limitations on the innovation side, the launch prices of DAA treatments posed significant access barriers. Sofosbuvir—the backbone of hepatitis C treatment to date—was launched in 2013 and priced at USD 84,000 for a standard 12-week course of treatment, despite having received over USD 60 million in public funding for its development. Daclatasvir, a DAA combined with sofosbuvir as the most widely used treatment—was launched in 2015 at a price of USD 63,000 for a treatment course [12,13]. These prices sparked considerable controversy and debate over the affordability and accessibility of hepatitis C treatment globally, leading to the rationing of treatment even in HICs, in addition to the lack of access in the majority of LMICs [8,10,13,14]. Between 2014 and 2018, most treated patients were in HICs, despite the fact that LMICs accounted for 89% of all HCV infections [4].

As a partial response, both Gilead and Bristol-Myers Squibb (BMS), the patent holders for sofosbuvir and daclatasvir, respectively, engaged in voluntary licensing agreements for the production and distribution of generic versions in certain countries, particularly low-income countries (LICs) and lower-middle-income countries (LoMICs), leading to an increase in treatment access because of the lower cost of generics [4]. While Gilead took a bilateral approach to their voluntary licenses, BMS opted to license to the Medicines Patent Pool (MPP), an international organization created to increase equitable access to innovative medicines through public health-oriented voluntary licensing and technology transfer [15,16]. However, as is often the case with voluntary licenses, most upper-middle-income countries (UMICs) and HICs were left out of the scope of the countries that could have access to the generic medicine produced under those licenses, and DAAs remained very expensive where generics were not available as those countries relied on price negotiations with the originator companies [3,7,10,11,13]. For example, in 2019, the median originator price per 12-week course of sofosbuvir was USD 40,500, and of daclatasvir, USD 27,000 [17].

Challenges regarding access due to high prices, as well as limited indication for some populations, led to the search for the development of new medicines that could address some of the problems associated with access to HCV treatments [7,11]. In previous research, we assessed the effectiveness of non-commercial innovation models, particularly those led by Product Development Partnerships (PDPs), focused on developing new medicines for neglected diseases [18]. An open question remained about the feasibility of expanding such models to disease areas with higher commercial interest, in which commercial partners could be less willing to contribute to public–private partnerships. In this article, we aim to contribute to filling this research gap by analyzing a collaborative effort initiated in 2016 led by a PDP for the development of ravidasvir, a new chemical entity (NCE) for the treatment of hepatitis C, a highly lucrative market. Following a novel conceptual framework (see methodology below), we analyze the innovation model, examining the actors involved, ways of securing resources (knowledge, financing, manufacturing capacity, and relationships), organizational practices (knowledge management, access strategies, and transparency), and outcomes.

## 2. Methodology

This publication is part of a broader research project that aims to understand whether and how alternative models of pharmaceutical research and development (R&D) can better meet the global public interest. We define an ‘alternative model’ of pharmaceutical innovation as an initiative that funds, implements, or facilitates R&D in a manner that differs significantly from the traditional market-driven innovation model and with the aim of better meeting global public interest objectives such as missing innovation or affordability.

Within the project, we developed a novel conceptual framework to analyse the pharmaceutical innovation system drawing from the study of Complex Adaptive Systems (CAS), yielding a Complex Adaptive Pharmaceutical Innovation System (CAPIS). We conceptualized the CAPIS as a combination of actors, resources, and the rules and norms that shape their interactions. We identified four kinds of resources needed to conduct R&D: knowledge, financing, manufacturing capacity, and relationships (a more detailed description of the framework is available upon request). This framework is used for the analysis of the case study presented here.

The case study methodology was utilized as a strategy to develop an in-depth understanding of the factors that influenced how new or alternative models fared in the pharmaceutical innovation system. Following an initial selection of 140 alternative R&D initiatives [19], the research team developed the following criteria to select three case studies for more in-depth examination: geography, disease area, organizational form, evidence of success, degree of the existing literature, information accessibility, and uniqueness. Based on these criteria, the development of the drug ravidasvir was selected (in addition to cholera vaccines and hospital-based CAR-T therapy).

This study employed qualitative research methods. First, we conducted desk research to gather background information about the project available on the organizations’ websites and the grey literature (e.g., news articles, press releases, etc.). Second, we conducted a literature search in the English language using the keyword “ravidasvir” in major databases, including PubMed, Global Index Medicus, and Google Scholar, from the earliest available literature until December 2023. Finally, we conducted semi-structured interviews with key actors involved in the project. Participants for the interviews were selected through purposive sampling, considering their relevance to this study’s objective and their role in the development of ravidasvir. We conducted 12 interviews, with the participation of 15 individuals. Interviews were conducted by at least one of the authors either through an online platform (Webex, version 44.9.1.30809) or in person when possible and lasted 60–90 min each. In-person interviews were audio recorded using the software Otter (version 3.59.240923.991). Transcriptions from the Webex and/or Otter recordings were created with the assistance of Otter or Microsoft 365 edition of Word (version 16.89.1). We used thematic analysis to analyze data based on the conceptual framework developed by the project. Interviews were anonymized, and quotes were edited for length and clarity. A list of interviewees is available below (with names listed according to consent from the interviewees), as well as a list of contacted organizations that either did not respond to our request, did not agree to participate, or were not available for an interview (Table 1). Interviewees were given the opportunity to provide comments on an earlier version of the manuscript.

## 3. Results: A Collaborative Innovation Model for the Development of Ravidasvir

### 3.1. Actors: Mission-Oriented towards Providing Affordable Medicines

The development of ravidasvir involved several actors, and its mid to late development was led by the Drugs for Neglected Diseases initiative (DNDi), a non-profit public–private product development partnership (PDP). While there is significant variation in how they operate, a PDP is usually a non-profit organization that acts as a ‘system integrator’ by bringing together academic, government, industry, and philanthropic actors to jointly develop new health technologies for unmet health needs [20,21]. For the development of ravidasvir, DNDi partnered with a private company based in Egypt, Pharco Pharmaceuticals, and with the governments of Malaysia and Thailand. Other actors also became involved, particularly in registration, manufacturing, and distribution, as will be described below.

DNDi was established in 2003 with a focus on the research and development of drugs for neglected tropical diseases (NTDs) and other diseases that primarily affect populations in lower-income settings, for which there is little financial incentive for pharmaceutical companies to invest in drug development. The organization’s initial scope—neglected diseases—was later extended to the broader concept of neglected patients [22].

*“As one of the potential expressions of the new extended mandate of DNDi, which is not anymore neglected disease, but neglected populations, HCV was identified as typically an area where neglected population was more the issue”*.

(I_9)

The motivation of DNDi to become involved in developing a treatment for hepatitis C was two-fold. First, the new treatments that were becoming available in 2013–2015 had been mainly developed for populations in HICs. The development of ravidasvir aimed to provide better treatment options for neglected populations, such as people infected with genotypes more prevalent in LMICs, people who use drugs, and people on treatment for HIV, a significant co-infection with HCV, who are usually not included in the target population of commercial clinical trials [7,8,10,11] (I_2, I_4, I_6, I_11).

*“The project was framed to address the needs of neglected populations with hepatitis C; the drug users, the drug interactions with HIV treatments. (…). So again, tailoring to those populations who are the more vulnerable populations”*.

(I_6)

Second, the prices of the new treatments were exorbitantly high, leading to limited global availability. Therefore, DNDi decided to develop a treatment option that would be available at an affordable price [8,10] (I_2; I_3; I_4; I_11). Ravidasvir could introduce additional competition to the HCV DAA market, potentially decreasing the prices of hepatitis C treatments overall (I_3; I_6; I_11).

*“The decision was to see if they could develop quickly another drug, and together with partners that would agree to offer a very competitive price close to the cost of goods, to put pressure on the markets where the prices of the treatments offered by big companies are much too expensive, to push the other companies to align towards the price of ravidasvir”*.

(I_3)

Pharco Pharmaceuticals, founded in 1983, was one of the leading pharmaceutical generic manufacturers in the Middle East and Africa region. The company, in addition to conducting clinical trials for ravidasvir in Egypt, was responsible for supplying the drug for clinical trials in other countries, developing the finished dose form for registration, and engaging in technology transfer agreements for production and distribution [8,10]. Pharco includes access as part of its mission, including the delivery of medicines that are affordable globally [23,24], and wanted to position itself as an important actor in global health:

*“At Pharco we are saving lives. Through innovative solutions, we can deliver the most affordable and effective treatments for all patients, everywhere”*.

(Dr. Sherine Helmy, CEO, Pharco Corporation) (https://pharco.org/about.html, accessed on 27 April 2024)

*“There was a moment in which the hepatitis medicines that came to market were very expensive, and there was a lot of concern about access in LMICs. There was a perception that Pharco was going to do things differently, wanting to be the ‘saviors’ of the hepatitis C space, as Cipla had been several years back in HIV. They seemed keen to partner with the public health community. They were seeking to position themselves as a leader in access in hepatitis C working closely with DNDi”*.

(I_12)

The governments of Thailand and Malaysia, both UMICs initially excluded from accessing generic DAAs produced under voluntary license agreements, were also involved in the development process, especially by sponsoring clinical trials, with the aim of having better-suited and more affordable treatment options for their populations and potentially beyond [8,10,25,26]. The government of Malaysia was also involved in fostering the registration, manufacturing, and distribution of ravidasvir, going beyond its clinical development. Next, we analyse further the role of the different actors involved in the development process, as well as in manufacturing and distributing ravidasvir, by identifying the different resources involved in the project.

### 3.2. Resources: Knowledge, Manufacturing, Financing, and Relationships

Following our conceptual framework, we analyzed four types of resources mobilized for the development of ravidasvir: knowledge, manufacturing, financing, and relationships.

The initial knowledge for the development of ravidasvir came from a licensing agreement with its initial developer, Presidio Pharmaceuticals, a biopharmaceutical company based in San Francisco, California, in the United States. Presidio played a significant role in advancing ravidasvir through preclinical studies and early clinical trials, including the completion of phase 1 clinical trials in 2012 [10]. In 2013, Presidio Pharmaceuticals and the multinational company Boehringer Ingelheim collaborated on a phase 2 combination trial as part of the development process for ravidasvir (ClinicalTrials.gov ID NCT01859962). The collaboration aimed to evaluate the effectiveness and safety of combining ravidasvir with other agents developed by Boehringer Ingelheim. The collaboration did not go further, reportedly because of the low efficacy of the combination drug being developed by Boehringer Ingelheim (I_5). It should be noted that ravidasvir is designed to be used in combination with other DAAs, and was later tested to be used in combination with sofosbuvir.

Following the decision to work on hepatitis C, DNDi assessed all drugs under development, as in-licensing an existing candidate drug can expedite the process, and decided to approach companies that were in earlier stages of development, as those were more likely to be willing to work with them (I_5). Smaller companies also tend to be more open to partnerships with PDPs who can contribute resources to R&D, as they have less capital available to invest in clinical studies (I_5).

*“And that is when it was decided to approach companies developing drugs, but not the frontrunners, and see whether they were willing to work together through licensing. Big pharmaceutical companies were approached as well, to see if they had plans to conduct studies in developing countries, but as expected, none of them were doing that for lack of markets, and they were just not interested in engaging in a partnership targeting populations in those countries”*.

(I_5)

Presidio, the initial developer, was likely to reach the market much later than other companies developing DAAs. Particularly for high-income markets, the molecule did not attract interest from large pharmaceutical companies (I_3; I_12). However, it attracted the attention of developers from developing countries. In 2014, Pharco Pharmaceuticals obtained an exclusive license for development and potential commercialization in Egypt and the MENA region. Simultaneously, Ascletis, a biopharmaceutical company based in China, secured an exclusive license for development and commercialization in China. The development process of ravidasvir by Ascletis took place separately from the collaboration involving Pharco and is beyond the scope of this study. However, it is worth mentioning that it was only tested for use in genotype 1 [27] (I_2; I_9).

*“Ascletis got the license for ravidasvir in China, and then it was driven by a company led by someone returning from the US, so they were kind of going for high income, high profits. So, it’s a different access strategy”*.

(I_9)

Following the licensing agreement, Pharco Pharmaceuticals conducted a phase 3 clinical trial with ravidasvir in combination with sofosbuvir in Egypt. At the time, Pharco was already producing sofosbuvir since the initial decision to reject Gilead’s patent application in Egypt led to a number of voluntary license agreements in the country [28]. These trials aimed to assess the safety and efficacy of the combination, particularly for the treatment of genotype 4, which is prevalent in the MENA region, and showed high efficacy rates [7,10].

In 2016, Presidio Pharmaceuticals licensed ravidasvir to DNDi for LMICs not included in the previous licenses, keeping the rights for HICs. It is worth noting that the license had the option to be extended to HICs, which was not exercised due to patent rights on sofosbuvir in HICs, which acted as a disincentive to identify an industrial partner to register and commercialize ravidasvir in HICs, as well as lack of funding to conduct clinical trials in those countries [8,10] (I_2, I_4). Therefore, the commercialization rights of ravidasvir in HICs remained with Presidio.

In the same year, Pharco signed an agreement with DNDi to supply both sofosbuvir and ravidasvir for clinical trials and, in partnership with DNDi and the governments of Malaysia and Thailand, initiated a phase 2/3 trial in those two countries [10,26,29]. The clinical trials were conducted in two stages, from 2016 to 2021, to assess the efficacy of the combination treatment ravidasvir/sofosbuvir across genotypes 1, 2, 3, 6, non-cirrhotic and cirrhotic patients, and HCV mono-infected and HCV/HIV co-infected subjects (STORM-C-1, Clinicaltrails.gov ID NCT02961426) [11] (I_4). The trial showed that the ravidasvir/sofosbuvir combination was safe and effective in curing hepatitis C for 97% of the participants, including hard-to-treat patients [7,11].

The governments of Malaysia and Thailand played important roles in the development of ravidasvir. Their involvement in the project was multi-factor and revealed the importance of previous relationships. First, the Ministry of Health (MoH) Malaysia is a founding member of DNDi and has demonstrated a longstanding commitment to the principles of the organization [29]. Second, Malaysia had a strong political commitment to hepatitis elimination [26]. Third, Malaysia has a history of using TRIPS flexibilities, including compulsory licenses, which facilitated the process of issuing a compulsory license for sofosbuvir, the combination drug used with ravidasvir [11]. Fourth, as an upper MIC, Malaysia is often left out of companies’ strategies to facilitate access to medicines, such as voluntary licenses or donations, and wants to invest more in R&D [26] (I_3; I_9). Thailand’s involvement came from previous collaborations with DNDi, a commitment to eliminating hepatitis C, and a will to invest more in R&D (I_3; I_9). Experts from both MoH directly participated in designing the protocols for the clinical trials [8]. A small contract research organization (CRO) based in Thailand was hired to support the monitoring of the trials, storage of the samples, data management, and statistics for the trials being conducted in both countries (I_3). While the Malaysian government had, and still has, a very active role in the project, the Thai government was less involved (I_3; I_9).

*“In the case of Malaysia, because the Director General of the Ministry of Health of Malaysia is a statutory board member of DNDi, he was very early exposed to the discussion around HCV. (…) I think those elements, HCV elimination commitment, an upper middle-income country, and then the fact that it’s a country that has no question on its ability to invest in R&D. I think it was the same in Thailand. But in Thailand, there was less exposure to DNDi work because they were not on the board of DNDi. So, they were less understanding and empathetic with the project. That is why in the end, it was a different commitment from the Ministry of Health of Malaysia and the Ministry of Health of Public Health in Thailand. Yet both saw the opportunity here to do some R&D on a disease that was important for them”*.

(I_9)

As a PDP, DNDi does not have in-house manufacturing facilities, which is accomplished through partnerships. Pharco is the main industrial partner for the registration, manufacturing, and distribution of ravidasvir, and from the beginning of the collaboration with DNDi, it committed to engaging in licensing and technology transfer agreements with local partners in LMICs to increase global availability (see section below on access policies).

Finally, regarding financing, the clinical trials were co-sponsored by the Malaysian and Thai Ministries of Health and co-financed by the Médecins Sans Frontières (MSF) Transformational Investment Capacity (TIC) initiative [7,8,11,30], which aimed to invest in projects that might improve outcomes for patients and/or the of ability MSF to provide medical assistance to people in need [31]. Information about the total amount of funding for the development of ravidasvir was not available in the public domain. According to interviewees, the Malaysian MoH invested around USD 15 million for the clinical trials (I_7), and about USD 10 million came from the MSF TIC program (I_6). There was also co-financing from the core budget of DNDi, as they could not find enough external funders to support the project (I_9). Data available in the financial reports for DNDi from 2015 to 2022 shows expenditures in the Phase 2/3 trial and registration costs for ravidasvir of around USD 14.2 million [32]. Therefore, funding sources for the development of ravidasvir were mostly public and philanthropic. Nevertheless, Pharco’s significant in-kind contribution to supplying the drugs used in the clinical trials should be noted (I_2).

### 3.3. Organizational Practices: Knowledge Management, Access Strategies, and Transparency

Having looked at how the actors obtained the main resources for the development of ravidasvir, we now turn to an analysis of how organizations used those resources—that is, their organizational practices—under three themes: (i) knowledge management, including intellectual property (IP); (ii) access strategies, including pricing and availability; (iii) transparency, particularly of contractual terms and R&D costs.

The initial licensing agreements between Presidio, Pharco, and Ascletis are beyond the scope of this study and are not available in the public domain; however, it should be noted that they were exclusive licensing agreements covering a particular geographical scope. Exclusive agreements limit the number of manufacturers, while non-exclusive agreements allow for competition between different manufacturers and may reduce prices and increase availability, security of supply, and access. While the license between Presidio and DNDi is also not fully available, some of its terms are available and include the payment of royalties to Presidio from the commercialization of ravidasvir in countries where there is a patent in force and that all clinical data generated would be shared with Presidio, which could eventually be used by Presidio to support registration in HICs, where the company holds the commercialization rights for ravidasvir [10] (I_2). Royalties are tiered depending on the economic level of the countries where the final drug is sold (4 or 7%) and payable by DNDi and its commercialization partners [10,33]. In this section, we analyze the knowledge management practices after the license to DNDi, that is, regarding knowledge generated in the scope of the collaborative model.

After having licensed-in rights from Presidio, both Pharco and DNDi engaged in several agreements for the further development, manufacturing, and distribution of ravidasvir. The agreement between Pharco and DNDi, which is also not fully available in the public domain, includes a clause for Pharco to supply the medicine at affordable prices, including for conducting clinical trials, and to engage in technology transfer agreements in other countries, particularly in LMICs [10] (I_2). In 2016, there was an announcement that the ravidasvir/sofosbuvir combination would not cost more than USD 300 for a 12-week treatment course in Malaysia, while the originator price of the sofosbuvir/daclatasvir combination was USD 75,000 for the same period in the country [8,10,13,34].

*“In the DNDi-Pharco agreement, Pharco was okay with sharing this technology so that it could be used and spread in other countries as needed. It’s one of those few companies that are still family-owned, with an access-oriented mission, and that is why you get a different centre of communication”*.

(I_2)

In 2017, Pharco, encouraged by DNDi [10], licensed its rights to ravidasvir to the Medicines Patent Pool (MPP), a global health organization backed by the United Nations with a mission to increase access to medicines in LMICs by pooling intellectual property rights through voluntary license agreements to facilitate generic manufacturing and the development of new formulations [35]. Pharco turned the exclusive license it had negotiated with Presidio into a non-exclusive license, allowing other manufacturers to commercialize in the countries included in that license. However, so far, no sublicenses have resulted from this license. The reasons might include the openness of DNDi and Pharco to licensing agreements outside of the MPP, patent barriers related to the combination drug sofosbuvir, or the availability of cheap generic versions of other treatment options.

*“There were commercial challenges of doing it because of the sofosbuvir issue. Companies were wondering what to do with this new drug [ravidasvir] since it could not be used on its own and it required the use of sofosbuvir. And most of the countries that were in the MPP license eventually would get access to generic daclatasvir already, so there was a very limited rationale for developing ravidasvir, which would have been a big investment when you could get very cheap daclatasvir”*.

(I_12)

The agreements with the governments of Malaysia and Thailand did not involve the licensing of the patent rights but were partnership agreements for the clinical trials and the management of its data. The agreements are also not fully available, but according to one interviewee, they include a clause that none of the partners can apply for data exclusivity rights over these clinical data (I_2). Data exclusivity is a type of protection granted in some countries for a period of time to the original market authorization holder, during which generic manufacturers cannot rely on clinical data submitted to a regulatory agency in their own applications, therefore delaying the availability of generic medicines. In the traditional pharmaceutical innovation model, companies usually apply for data exclusivity, allowing them to limit generic competition further. The fact that neither DNDi nor its partners hold data exclusivity rights over ravidasvir reduces potential barriers to generic manufacturing.

While ravidasvir is still under patent protection held by Presidio, the licensing agreements with Pharco and DNDi cover most LMICs: 139 countries, accounting for 85% of people with hepatitis C [36]. Both actors are engaging and open to engaging in non-exclusive licensing agreements with interested manufacturers. The fact that IP is not a barrier for ravidasvir was pointed out as being very valuable for the uptake of the medicine in different countries (I_9; I_11; I_12):

*“When you come to ministries of health and say, ravidasvir, there is no license problem for it, you can have it. That is something they value a lot”*.

(I_9)

Regarding access, ravidasvir is currently registered in Malaysia, Egypt, and China [37]. In June 2021, the National Pharmaceutical Regulatory Agency (NPRA) of Malaysia granted a conditional registration for ravidasvir, and full approval was granted in February 2024 [38,39]. It was the first time that the Malaysian regulatory agency registered an NCE that was not previously evaluated by a stringent regulatory agency (I_2, I_6).

Ravidasvir achieved recognition and global importance by being included in the World Health Organization’s (WHO) Essential Medicines List (EML) in 2023 at the request of the Malaysian government [37,40]. This was the first time that a medication was included in the EML at the request of the government (I_1; I_11). The inclusion of ravidasvir in the EML provided countries with additional treatment options to treat their populations, including the possibility of increasing security of supply because of the possibility of local production.

*“It was opening the door for increasing access, decreasing prices through competition across the different molecules. It might not impact all countries, but there is the potential of this new treatment option having an impact at least in some countries where it can increase access and may be preferred to other options. (…) And it is important to increase the number of options and also to have essential medicines that are much closer to the countries on the same geographic areas, for which there are not only a few sites of production, and the procurement and logistics are not highly dependent on a long chain”*.

(I_11)

In 2022, the first patient in Malaysia was treated with the combination of ravidasvir/sofosbuvir [30]. Malaysia started using ravidasvir as part of its test and treat strategy soon after it obtained regulatory approval in the country. That was due to the development of an access strategy during the development process, which accelerated the product uptake in the country (I_9).

*“Malaysia used the clinical trials to design a very aggressive access strategy. (…) Very early on, there were discussions about access; once the clinical trial idea was started, they very quickly sat and discussed access: once the product exists, what are we going to do? How are we going to do that? The same did not happen in Thailand, and ravidasvir is still not currently available in the country”*.

(I_9)

For the registration, manufacturing, and distribution of ravidasvir in Malaysia, there was a partnership with a Malaysian pharmaceutical company, Pharmaniaga Berhad, affiliated with the Malaysian military [8,11,24]. It had a trilateral agreement with DNDi and Pharco, which involved a sublicence from DNDi and a technology transfer agreement from Pharco for local production in Malaysia [8]. The company invested USD 2.2 million in preparing the regulatory dossier and its manufacturing facilities [41] (I_3; I_9). However, despite being the registration holder in the country, as of this writing Pharmaniaga was still importing from Pharco to supply ravidasvir in Malaysia (I_2). It was planned for Pharmaniaga to start local production, but production is still not taking place because of market dynamics and changes in the company’s leadership (I_2; I_9).

*“The leadership was very access-minded, really open, and very much looking at the future. And they were really visionary in understanding the impact that ravidasvir could have on them. They really saw that as an opportunity to learn how to register a new chemical entity. They saw that as an opportunity to promote their own brand. Again, moving forward from being just a generic manufacturer”*.

(I_9)

Aiming at making ravidasvir more globally available, Pharco and DNDi engaged in strategic partnerships aiming to increase access, reduce prices through economies of scale and ensure security of supply by increasing the number of producers. Pharco, the main industrial partner in the project, is responsible for taking the lead for registration and possible technology transfer agreements in different countries, while DNDi is playing the role of connecting them with partners that might be interested in becoming involved in the negotiations and licensing the rights to the medicine in DNDi territory (I_9, I_10). While the national partner is responsible for covering all costs, DNDi is still committed to providing regulatory support for the registration, using its institutional resources to cover its staff costs (I_10).

In Latin America, there are agreements for technology transfer, registration, production, and distribution of ravidasvir in Argentina and Brazil. The mix of opportunities and the presence of partners historically close to DNDi, with a shared vision on access issues, were deciding factors in the partnerships in the region (I_10). In Argentina, the agreement is with Laboratorio Elea Phoenix, a national private company that has collaborated with DNDi previously [8,38] (I_2). The regulatory dossier was submitted in 2022 by Laboratorio Elea, with support from Grupo Insud and Fundación Mundo Sano, both philanthropic foundations [8,30]. However, the technology transfer agreement has not started yet, as Elea reportedly, has hesitated to make all the necessary investments in light of unclear demand for ravidasvir, given other drugs currently available for the treatment of hepatitis C (I_2).

In Brazil, the state-owned company Farmanguinhos/Fiocruz, a founding member of DNDi, signed in July 2023 an initial agreement with DNDi and Pharco to register ravidasvir in the country and have it approved to be used in the public health system, with DNDi providing clinical data and technical support for the regulatory dossier, and Pharco providing the drug for additional local clinical trials that might be required for registration. Once the registration is completed, the technology transfer agreement with Pharco for local production in the country is expected to be resumed [42,43] (I_8, I_10).

Finally, in March 2024, Pharco and DNDi signed an agreement with Mahidol University, a public university in Thailand, for the introduction of ravidasvir in the country (no further details are available in the public domain) [39,44].

**Figure 1:**
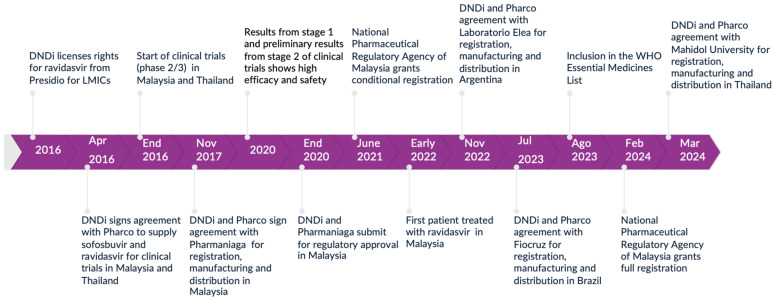
Ravidasvir’s project timeline.

The figure below provides a timeline with the main milestones in the development process and scale-up of ravidasvir, starting from 2016 with the licensing agreement between Presidio and DNDi (Figure 1).

### 3.4. Outcomes

The collaborative model led to the development of an effective, easy-to-use, and affordable treatment for HCV, demonstrating the feasibility of developing medicines through alternative innovation models even in areas of high commercial interest. It also had other important outcomes in developing R&D capacities in LMICs and potentially acting as a market shaper. However, internal constraints and changes in the external context over the duration of the project, especially the strategy of companies with pre-existing products to make generic versions available in many LMICs, reduced the potential public health impact of the drug. At the time of writing, there were ongoing developments aiming to increase the demand for ravidasvir, particularly by reducing treatment time and price. It is worth recalling that ravidasvir is a new chemical entity, and the raw material for its production had no previous commercial demand, therefore contributing substantially to the cost of the final product (I_4).

**Figure 2:**
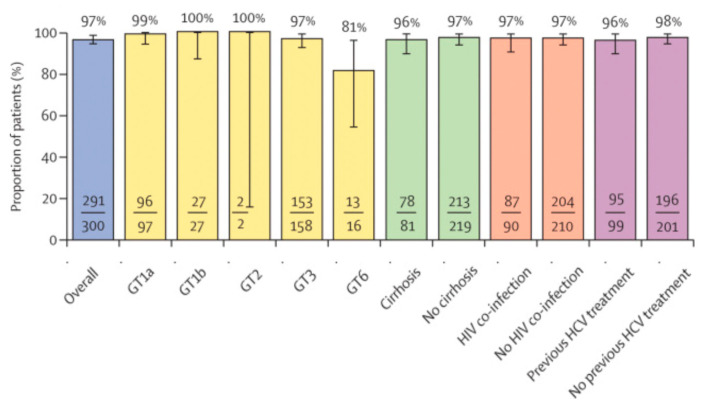
Ravidasvir’s efficacy. Source: [7].

Regarding efficacy, the ravidasvir+sofosbuvir combination has demonstrated similarly high efficacy to other regimens, with an overall cure rate of 97%, including for genotype 3, which is more prevalent in LMICs, people co-infected with HIV and people with cirrhosis (Figure 2) [7,37,45]. However, it should be noted that genotype 6 showed lower efficacy (81%), and there were only two people with genotype 2 in a total of 300 included in the trial [7] (for further information on the efficacy of various competing products, see [45,46]). It provides a highly effective additional treatment option for people with hepatitis C, as well as being an important alternative in case of resistance to other treatments (I_4).

It should be noted, however, that challenges that occurred during the development process of ravidasvir reduced clinical data currently available, potentially limiting its use. Currently, ravidasvir has demonstrated the potential to be pangenotypic but lacks clinical data regarding genotype 5 and has limited data for genotype 2. Clinical trials were originally planned to occur in South Africa for genotype 5, but they did not happen due to budget limitations (I_4; I_6; I_9). Nevertheless, ravidasvir, when used in combination with sofosbuvir, has been included in the WHO EML as pangenotypic [40], and national regulatory agencies may act the same, particularly if genotype 5, which is very rare, is not significantly prevalent in their population.

*“The dossier is enough to show efficacy and safety on genotypes 1, 3, 4 and 6. However, when a regulatory agency registers the product in the country, it is ultimately sovereign. In Malaysia, it is pangenotypic because it covers all the genotypes existing there. The WHO EML decided genotype 5 is a very rare genotype. Genotype 2 is very easy to treat, and there is data on the efficacy on genotype 2 in vitro; what is missing is enough clinical data”*.

(I_9)

Ravidasvir demonstrated no significant drug–drug interactions with antiretrovirals commonly used for the treatment of HIV, meaning that no adjustments in the treatment are needed for this population [7]. However, current data available for other DAAs, particularly for the combination sofosbuvir/daclatasvir, also shows no significant drug interactions with currently recommended HIV drugs (as other HIV drugs that had higher interactions with DAAs were replaced), reducing that particular use for ravidasvir (I_6).

Regarding pricing, the offered price for ravidasvir in the public sector in Malaysia is USD 3.59 per tablet, or USD 300 per 12-week treatment [37], which is a very small fraction of the price of other DAAs at launch, recalling that ravidasvir is an NCE. Sofosbuvir is available in Malaysia for about USD 30, making the ravidasvir+sofosbuvir treatment priced at around USD 330 in the country [37]. However, market dynamics considerably changed since the project first started, particularly reducing the price of its main competitor, daclatasvir, leading to reduced demand for ravidasvir (I_6).

Since ravidasvir has to be used in combination with sofosbuvir, it is impacted by sofosbuvir’s price and availability. Gilead, the originator of sofosbuvir, made a strategic market decision to sell their competing treatment (sofosbuvir/velpatasvir) at a lower price than sofosbuvir alone (I_2, I_6, I_12). For example, in 2019, the median originator price for sofosbuvir alone was about USD 40,500, higher than the price for the sofosbuvir/velpatasvir combination (USD 34,381) [17]. Gilead’s strategies might limit the use of ravidasvir in countries where sofosbuvir is still under patent protection and is therefore costly or where Gilead has chosen not to sell sofosbuvir alone at all.

Gilead’s pricing strategy might have also had an impact on the current price of daclatasvir, another drug that has to be used in combination with sofosbuvir and that is in the same class as ravidasvir. Gilead’s strategies might have influenced the decision of BMS to eventually stop the commercialization of daclatasvir in 2020, as they would have to lower the price too much to remain competitive (I_2, I_6, I_12). With the announcement that BMS, the originator, would no longer enforce its patent rights in most countries, generic companies could also supply daclatasvir in countries previously excluded from the voluntary licenses without fear of litigation from BMS and they began to supply these generics [1]. It should be noted that the main patent for daclatasvir was due to expire in 2027 [47], and in 2019 the median originator price for daclatasvir was USD 27,000 per 12-week course [17], which would have made ravidasvir an extremely affordable alternative to daclatasvir. However, the current availability of generic versions of sofosbuvir and daclatasvir in many LMICs at prices as low as USD 60 makes it less attractive to replace daclatasvir with ravidasvir, reducing the total demand. Nevertheless, that price is not available in all countries, as can be exemplified by Brazil, a UMIC where the price for daclatasvir alone in 2023 was almost USD 500 per 12-week course [48].

*“People were starting to get access to generics of sofosbuvir and daclatasvir, but mostly in low-income countries and the countries included in the voluntary license of Gilead for sofosbuvir and the BMS-MPP license for daclatasvir. But some upper-middle-income countries were struggling for access, as they couldn’t access the generics from those voluntary licenses. In the countries where there is still a commercial market for Gilead, there was no option to use sofosbuvir with daclatasvir, as sofosbuvir alone was not available, or it was super expensive. So ravidasvir was of interest. The big game changer was BMS leaving the market. If there was still a patent on daclatasvir, we might have been in a completely different space”*.

(I_6)

Furthermore, it should be noted that the announcement of the target price for ravidasvir at USD 300 when the project started in 2016 might have had an impact as a market shaper, both for branded and generics alike [26].

*“Even if ravidasvir is not used very much today, the announced price in 2016, that ravidasvir would be available for USD 300, served as a benchmark to push Gilead to extend its licenses and reduce its price”*.

(I_2)

*“Following the announcement of the USD 300 for ravidasvir, Malaysia rapidly took this as a reference price, and while waiting for the development of ravidasvir, negotiated a price with a generic manufacturer of sofosbuvir for USD 300. This was the first impact of the announcement”*.

(I_4)

However, while ravidasvir might have had a role as a market shaper in some countries, it might not have significantly impacted the hepatitis C market as a whole, particularly in countries where multiple generic options were available for sofosbuvir and daclatasvir.

*“We knew that generic versions of sofosbuvir and daclatasvir were going to be coming in because of the existing voluntary licenses and we knew that the prices were going to be low. While we did not know how low it was going to get, that price [USD 300] was going to be beaten by the generics anyway and was very rapidly beaten, at least in countries where there was more than one generic registered and competing. The negotiated price now for the combination of generic sofosbuvir and daclatasvir is $60”*.

(I_12)

Additionally, there are initiatives in place to make ravidasvir more convenient for patients and potentially reduce its price. Ongoing trials led by the Malaysian MoH are exploring the potential benefits of reducing treatment time and dose, aiming to make ravidasvir more cost-effective. Currently, the standard treatment time is 12 weeks for non-cirrhotic patients, and trials are ongoing to reduce the treatment time to 8 weeks, with results expected in mid-2024 (I_4, I_7). The possibility of doing a fixed-dose combination to facilitate ease of use is also being explored (I_4). The inclusion of ravidasvir in WHO’s EML is also expected to increase the uptake of ravidasvir in other countries, increasing its volume and reducing its price (I_7). It was noted, however, that it usually takes four to five years to see the impact of the inclusion of the EML in countries’ uptake of a new medicine (I_11).

*“Once it is on the WHO essential medicine list, more countries will learn about this new treatment. But the question is whether they want to use it or not. So unless you give them the data regarding efficacy and effective treatment of certain genotypes in their country, or if you can bring down the cost much cheaper, or if you can reduce treatment time from 12 to 8 weeks, which is more convenient to the patient, they might not want to use it. So there is ongoing research to assess these options”*.

(I_7)

Regarding availability, ravidasvir is currently in use only in Malaysia, highlighting the need for further expansion to address the global hepatitis C burden. Registration processes are underway in key countries, including Brazil, Argentina, and Thailand. Successful registrations would significantly broaden the geographic availability of ravidasvir and could potentially reduce price by increasing volume [8].

The ongoing efforts in clinical trials, coupled with strategic pricing and availability initiatives, could contribute to the use of ravidasvir in combating hepatitis C on a global scale. However, the absence of international funding programs for hepatitis C was raised as a limiting factor for increasing treatment in LMICs, even with generic medicines available.

*“There is not a lot of funding for hepatitis, which has been problematic for the past 10 years or more. Because now, even if we have generics available, as there is no big donor funding, they are still not used much”*.

(I_6)

In addition to the successful development of ravidasvir, another important outcome of this collaborative endeavor is capacity-building in LMICs. It was the first time Pharco became involved in developing an NCE (I_3). It was also the first time that the Malaysian regulatory agency registered an NCE that was not previously registered in another country, increasing its capacity to become a reference for the registration of NCEs (I_2, I_6). In addition, Pharmaniaga became involved for the first time with the development and registration of an NCE (I_2, I_3) [11]. As put by one interviewee:

*This is not the mainstream. This is really an alternative stream full of learning”*.

(I_3)

*“It was very interesting for all of these partners based in LMICs because it was more than just getting access to an affordable drug; there was a whole capacity development aspect of it, which has been part of the project from the very beginning”*.

(I_2)

The development of capacities was also raised as an important factor for actors engaging in technology transfer agreements for the manufacturing of ravidasvir, as companies in LMICs are typically less frequently engaged in the development of innovative treatments.

*“These companies would be the market authorization holder for an innovative treatment, which is not a position they are typically in. It’s likely quite a recognition for these companies if they manage to be the innovator for a new chemical entity in the country”*.

(I_12)

The strengthened capacities in Malaysia were already instrumental in accelerating access to other health technologies, such as for COVID-19. As explained by one interviewee:

*“Later, when COVID hit the world, Pharmaniaga decided to submit an application for registration of a vaccine, the Sinovac, something that would never have been done if they had not registered ravidasvir because it was a new vaccine. And, as a generic manufacturer, they had zero experience with new products prior to ravidasvir. And that gave them the knowledge and the courage to do it. And it’s also the same with the regulatory agency, which set up a series of re-adapted or renewed processes for ravidasvir, such as conditional registration. And when they had to register the vaccines for COVID, they already had a pathway that they had redeveloped for ravidasvir. This is where the impact of this project is massive”*.

(I_9)

Despite all that, stronger support and recognition from global health institutions was raised by one interviewee as still very much needed for LMICs conducting product development.

*“We have a global health system that is constantly asking countries to get involved to invest. And here, we have a typical example of a country that has done that, Malaysia; they have invested, and they have taken responsibility for access. What do we get from the global health system? Only problems and criticisms. Oh, it’s not this. It’s not that, it’s the only thing they see. Global health being mostly a Western-driven agenda. And the conclusion is, in my opinion, unless you’re British, French, American, German, forget about it. The global health system will never support you”*.

(I_9)

Issues related to regulatory standards were particularly raised as an area where greater recognition of capabilities in developing countries is needed from global health institutions. Obtaining approval from a stringent regulatory authority (almost all based in HICs), as well as pre-qualification by the WHO, can be prohibitively expensive and impose a high burden on developers. As explained by one interviewee:

*“It’s not the quality; it’s that we can’t afford to do a pre-qualification with WHO or stringent regulatory authority; it’s too expensive. In a positive way, outside of this very stringent global health environment, there are a lot of opportunities in LMICs where you don’t compromise on quality and safety, and where you can still work with partners to develop new treatments and make them available. It doesn’t mean the regulatory environment is not stringent. It’s just not protectionist. So, it’s about identifying and developing those opportunities outside of that bubble. And that’s what we have done with ravidasvir”*.

(I_9)

## 4. Discussion and Conclusions

**Figure 3:**
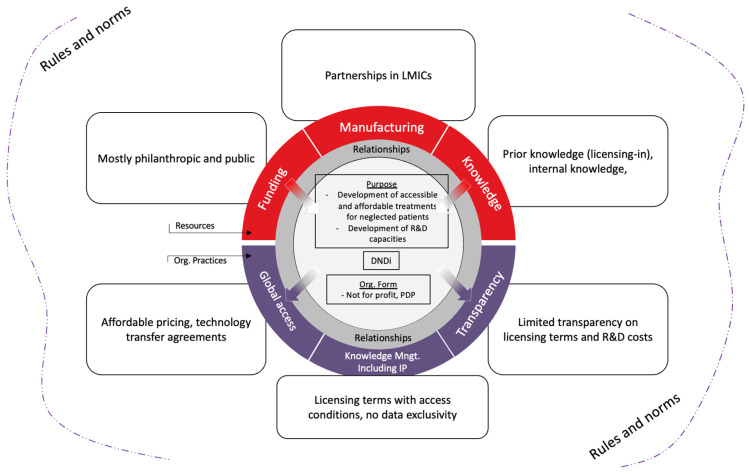
Ravidasvir’s innovation model.

By analysing the actors, resources, and organizational practices used to develop ravidasvir, we derived an innovation model summarized in the figure below (Figure 3).

The development of ravidasvir took the form of global collaboration, particularly engaging actors from the Global South. DNDi, a global PDP, was at the centre of the project, acting as a system integrator, pulling together actors and resources. Of particular importance was the previous relationship of the different partners with DNDi, which facilitated engagement in the project. Financing came mostly from a mix of philanthropic and public sources, with substantial private investments from Pharco. Knowledge came first from licensing-in prior knowledge from a private company (Presidio), and internal knowledge from DNDi and the MoH of Malaysia and Thailand was central in designing and conducting the clinical trials. Manufacturing is conducted through partnerships, particularly with Pharco, which is open to sharing its technology and know-how through transfer agreements with other partners in the Global South, also aiming to increase global access. All partners were previously committed to access principles, facilitating affordable pricing and knowledge sharing through licensing agreements, and not pursuing intellectual property/data protection. Transparency, however, is still limited. While there is some information about licensing terms, actual agreements were not made available in the public domain, and there is even less scattered information about R&D costs, prices, and availability of ravidasvir. Despite limited information about R&D costs, it is worth highlighting that total costs were in the 10s of millions USD, far from hundreds of millions or billions mentioned in some estimates available in the literature [49].

The innovativeness of the model was highlighted by one interviewee below:

*“It is clearly a model that does not repeat the usual practice in which you have commercialization and also development by a private company, usually from high-income countries, and then it is also the same company that is commercializing the molecule, often not considering its affordability. In this case, there was an attempt by several entities to develop a molecule to improve the accessibility to this new treatment. The model was exceptionally interesting in itself because the molecule was designed from the beginning to bypass some of the access barriers, in which the final result should be a molecule with an accessible price. How many times do we see this? It is exceptional and should be replicated”*.

(I_11)

The development of ravidasvir by DNDi differs in important ways from other projects. As mentioned earlier, DNDi typically works with neglected diseases for which there is limited or no market. Hepatitis C has a large market in HICs as well, with about 3.7 million people being treated with DAAs between 2014 and 2022 [4]. By the end of 2019, originator sales of DAAs had reached USD 82 billion [17]. Being a highly profitable area, it was expected that collaboration from large for-profit companies would not be the same as in less competitive areas.

*“Usually for neglected tropical diseases for which there is no market at all, we often partner with the pharma sector because this is serving the needs that could be marketing in Africa, corporate social responsibility. So, it is really easy to align with pharma in non-competing areas. Of course, for the development in hepatitis C, it was not necessarily something we could partner with Big Pharma because the strategy of low prices entering into competition with other drugs and for which the pharma sector is expecting returns and revenues, is not in their interest”*.

(I_3)

That difference impacted the strategies usually adopted by DNDi, including the choice of partners. In this case, both industry and government partners had no prior experience in developing NCEs. It was the first time that DNDi applied to register an NCE without partnering with a major pharmaceutical company [11]. It is also worth noting that, differently from other projects, DNDi had to use its core budget for the development of ravidasvir, in view of difficulties to secure external direct funding (with the exception of MSF).

In addition to developing the R&D capacities of the actors involved, the involvement of actors from the Global South in the collaborative development of ravidasvir and the willingness to engage in technology transfer agreements for local production have been highlighted as important factors for its potential uptake by developing countries, as well as a driver for further South–South cooperation for other diseases [24] (I_8, I_9).

*“There is also one thing that can be a differentiator, which we think is quite important, is that it comes from a successful development of an NGO, and secondly, it is in the axis of collaboration that strengthens countries in the Southern Hemisphere with information and technology exchanging, which will strengthen regional policies for other diseases”*.

(I_8)

This case also highlights the so-called ‘middle-income trap’ in global health policies. Ravidasvir is particularly of interest to UMICs, which are not eligible for most of the international procurement mechanisms and are frequently excluded from voluntary license agreements [26]. Currently, generic versions of the sofosbuvir/daclatasvir combination are available for USD 60 for eligible countries [50], which excludes particularly UMICs. For example, in Thailand, a UMIC, while generic DAAs are available, the prices remain at USD 700–1000 per 12-week course [37]. Therefore, ravidasvir can be particularly useful in MICs where DAAs are still expensive.

*“Malaysia is in the middle-income trap. Not rich enough to pay, even rich countries have issues paying USD 90,000 dollars per patient. And not poor enough, as a middle-income country, to be eligible for voluntary licenses and international procurement mechanisms”*.

(I_7)

The development of ravidasvir demonstrated the feasibility of the PDP model in developing novel medicines also for diseases with high commercial interest, providing an effective and affordable treatment option for neglected populations in developing countries. Being a highly competitive market, less collaboration from large pharmaceutical companies was expected, leading to the engagement of partners with less experience in the development of novel medicines, both private and public. That had important implications for leveraging and developing capacities, particularly from actors from the Global South, reinforcing that successful development of novel medicines can be accomplished through collaborations based in LMICs and without the traditional reliance on major pharmaceutical companies [11]. Nevertheless, it is possible that the engagement of less experienced partners, as well as reduced funding availability, resulted in extended timelines for the project.

Furthermore, competitive markets are characterized by the presence of multiple players vying for market share. Firms engage in strategic behavior to undercut competitors and gain an advantage in the market, as seen with Gilead’s pricing and licensing strategies regarding sofosbuvir. PDPs and other actors that usually operate in the non-profit sphere may be less familiar with and have fewer resources to adopt counter strategies proactively. In the field of neglected diseases, typically, the number of actors involved in product development is limited, and few competing products are available. As a result, the introduction of a new treatment often encounters minimal or no competition. In the case of hepatitis C, external factors rapidly altered the landscape since the development of ravidasvir first began. The gradual availability of low-cost generic pangenotypic treatments, with no significant drug interactions with HIV drugs, greatly reduced the potential demand for ravidasvir.

Finally, the collaborative development of ravidasvir highlighted the importance of engaging with private and public partners with common visions and goals, who are willing to work together, sharing knowledge and experiences towards a common public health objective. Previous engagement with DNDi and alignment with its mission was highlighted as important factors for the involvement in the development of ravidasvir and current efforts regarding technology transfer for local production and distribution.

*“The development of this new drug is the result of a partnership between public and private actors sharing the same public health objective from the very start: the development of an affordable medicine”*.

(Dr. Bernard Pécoul, founder, DNDi) (https://dndi.org/research-development/portfolio/ravidasvir-sofosbuvir/, accessed on 27 April 2024)

The collaborative development of ravidasvir and the commitment of various stakeholders showcase a model that could pave the way for future initiatives aimed at tackling not only neglected diseases but also beyond, including diseases areas with high commercial interest.

## Figures and Tables

**Table 1:** List of interviewees and contacted organizations.

Interviewees		
Organization	Position	Name
Drugs for Neglected Diseases initiative (DNDi)	Intellectual Property and Access Leader	Pascale Boulet
Drugs for Neglected Diseases initiative (DNDi)	Former R&D Director	Shing Chang
Drugs for Neglected Diseases initiative (DNDi)	HCV Access Leader	Graciela Diap
Drugs for Neglected Diseases initiative (DNDi)	Research and Development Director	Laurent Fraisse
Drugs for Neglected Diseases initiative (DNDi)	Head of Discovery and Partnerships, DNDi Latin America office	Jadel Kratz
Drugs for Neglected Diseases initiative (DNDi)	Director, DNDi Southeast Asia office	Jean-Michel Piedagnel
Drugs for Neglected Diseases initiative (DNDi)	Viral Diseases Cluster Director	Isabela Ribeiro
Drugs for Neglected Diseases initiative (DNDi)	Public Health Policy and Programme, HIV, HCV and Mental Health, DNDi Southeast Asia office	Chung Han Yang
Farmanguinhos, Oswaldo Cruz Foundation	Director	Jorge Mendonça
Farmanguinhos, Oswaldo Cruz Foundation	-	anonymous
Government of Malaysia	Former Director-General, Ministry of Health	Hisham Abdullah
Government of Malaysia	Health Counsellor, Permanent Mission to the UN	Nurhafiza Md Hamza
Médecins sans Frontières (MSF)	-	Anonymous
Medicines Patent Pool (MPP)	Director of Strategy, Policy, and Market Access	Esteban Burrone
World Health Organization (WHO)	Technical officer, Essential Medicines List team	Lorenzo Moja
**Contacted organizations (not interviewed)**		
HC Pharma, Switzerland		
Pharco Pharmaceuticals, Egypt *		
Presidio, United States of America		

* Despite not being available for an interview, Pharco Pharmaceuticals provided comments on an earlier version of the manuscript.

## Data Availability

The datasets presented in this article are not readily available to protect participant confidentiality, given the small number of people involved in the case. Requests to access the datasets should be directed to marcela.vieira@graduateinstitute.ch.
